# The prognostic value of IDO expression in solid tumors: a systematic review and meta-analysis

**DOI:** 10.1186/s12885-020-06956-5

**Published:** 2020-05-26

**Authors:** Sen Wang, Jia Wu, Han Shen, Junjun Wang

**Affiliations:** 1 Department of Clinical Laboratory Medicine, Jinling Hospital, Medical School of Nanjing University, Nanjing, 210002 China; 2 Department of Clinical Laboratory Medicine, Nanjing Drum Tower Hospital, Medical School of Nanjing University, Nanjing, 210008 China

**Keywords:** Meta-analysis, IDO, Solid tumor, Survival

## Abstract

**Background:**

Indoleamine 2,3-dioxygenase (IDO) is a rate-limiting enzyme in the metabolism of tryptophan into kynurenine. It is considered to be an immunosuppressive molecule that plays an important role in the development of tumors. However, the association between IDO and solid tumor prognosis remains unclear. Herein, we retrieved relevant published literature and analyzed the association between IDO expression and prognosis in solid tumors.

**Methods:**

Studies related to IDO expression and tumor prognosis were retrieved using PMC, EMbase and web of science database. Overall survival (OS), time to tumor progression (TTP) and other data in each study were extracted. Hazard ratio (HR) was used for analysis and calculation, while heterogeneity and publication bias between studies were also analyzed.

**Results:**

A total of 31 studies were included in this meta-analysis. Overall, high expression of IDO was significantly associated with poor OS (HR 1.92, 95% CI 1.52–2.43, *P* < 0.001) and TTP (HR 2.25 95% CI 1.58–3.22, *P* < 0.001). However, there was significant heterogeneity between studies on OS (I^2^ = 81.1%, *P* < 0.001) and TTP (I^2^ = 54.8%, *P* = 0.007). Subgroup analysis showed lower heterogeneity among prospective studies, studies of the same tumor type, and studies with follow-up periods longer than 45 months.

**Conclusions:**

The high expression of IDO was significantly associated with the poor prognosis of solid tumors, suggesting that it can be used as a biomarker for tumor prognosis and as a potential target for tumor therapy.

## Background

Indoleamine 2,3-dioxygenase (IDO) is an intracellular and immunosuppressive rate-limiting enzyme in metabolism of tryptophan to kynurenine [1]. Tryptophan is an essential amino acid in protein synthesis and many important metabolic processes and cannot be synthesized in vivo. The main metabolic pathway for tryptophan in mammals is the kynurenine pathway, and this pathway requires participation of members from the IDO family. The IDO family of genes includes IDO1 and IDO2. IDO1 has higher catalytic efficiency than IDO2 and is more abundant in tissues [2]. In this systematic review and meta-analysis, the term ‘IDO’ will refer to IDO1.

IDO can exert immunosuppressive effects through a variety of mechanisms. The high expression and activity of IDO leads to a large consumption of tryptophan in the cell microenvironment, which makes the cells in a “tryptophan starvation” state. Depletion of tryptophan causes T cells arrest in the G1 phase of cell cycle, thereby inhibiting T cell proliferation. The main metabolite of tryptophan degradation, kynurenine, also has a direct toxic effect on T cells and induces T cell apoptosis. Kynurenine is also a natural ligand for aryl hydrocarbon receptors. By activating aryl hydrocarbon receptors, kynurenine can regulate the differentiation direction of Th17/Treg cells, thereby promoting the balanced differentiation of Th17/Treg to Treg cells [3–5].

IDO plays an important role in a variety of disease processes such as chronic inflammatory diseases, infection, and cancer [4, 6–8]. Increased expression of IDO is observed in many types of tumors, including colorectal, hepatocellular, ovarian and melanomas [5]. Tumors with high expression of IDO tend to increase metastatic invasion and have a poor clinical outcome in cancer patients. IDO is considered to be a new target for tumor therapy, and inhibition of IDO activity by using IDO inhibitors can increase patient survival [9–11].

Although IDO-targeted tumor therapy strategies are currently being developed, the association between expression level of IDO in tumor tissues and prognosis of patients remains unclear. Therefore, we constructed this meta-analysis to explore the correlation between IDO expression and tumor prognosis.

## Methods

### Search strategy

The present systematic review and meta-analysis was conducted and reported according to the standards of quality detailed in the Preferred Reporting Items for Systematic Reviews and Meta-Analyses (PRISMA) statement [12]. Comprehensive and systematic search of published literature using the following database, such as PMC, Embase, and Web of Science (up to May 31, 2019). We used keyword such as: (“IDO” or Indoleamine 2,3-dioxygenase) AND (cancer or carcinoma or tumor or neoplasms) AND prognosis to search in the database. The retrieved information of relevant literature was downloaded and imported into the literature management software for further browsing and screening.

### Inclusion criteria

Studies included in this meta-analysis needed to meet the following inclusion criteria: 1) The included literature needed to provide appropriate prognostic indicators in evaluating the expression of IDO and prognosis of solid tumors, such as overall survival (OS), progression-free survival (PFS), disease-free survival (DFS) or relapse-free survival (RFS). 2) The included literature needed to provide hazard ratios (HRs) with 95% confidence intervals (CIs). 3) The included literature needed to provide criteria for defining IDO expression as positive and negative, or strong and weak expression.

### Exclusion criteria

This meta-analysis had the following exclusion criteria: 1) The type of literature was not a research article but the following types:reviews, case reports, letters, editorials, and meeting abstracts; 2) Animal experiments or in vitro experiments rather than patient-based clinical studies; 3) HRs and 95% CI were not directly provided in the study; 4) Research was not published in English; 5) Sample size was too small, less than 50; 6) IDO expression was not detected in tumor tissues.

### Data extraction

**Fig. 1:**
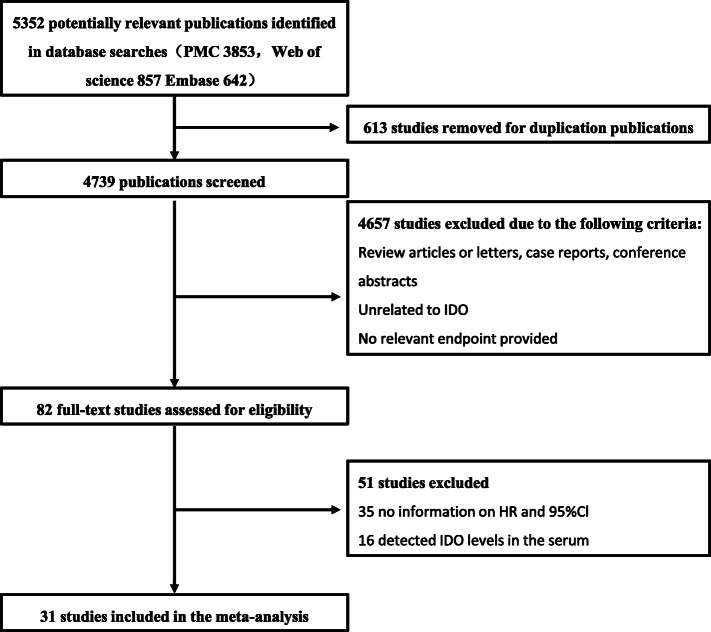
The flow chart of the selection process in our meta-analysis

The data extraction included in the studies were independently completed by two researchers according to the same criteria, and if there was inconsistency, a group discussion was conducted. This meta-analysis used two outcome endpoints: OS (overall survival) and TTP (time to tumor progression). Since PFS, DFS and RFS are similar outcome endpoints, we in this meta-analysis used the same prognostic parameter TTP to represent them. We extracted the following information from each study: first author’s name, publication year, country, cancer type, case number, study type, IDO detection method, cut off values for IDO expression, endpoints and HR. When the study provided HR for both univariate and multivariate analyses, we preferred results from multivariate analysis. The main features for these eligible studies are summarized in Fig. 1. Quality assessment for the included studies using the Newcastle-Ottawa Scale (NOS) [13]. According to the NOS system, the quality judgment for the studies were based on three parts: selection of study groups (4 points), comparability of study groups (2points), and outcome assessment (3 points). Studies with NOS scores above 5 were considered to have higher quality.

### Statistical analysis

Combined HR and 95% CI were used to assess the effect of IDO expression on tumor prognosis. HR > 1 and 95% CI did not overlap 1 indicating that overexpression of IDO had a negative impact on tumor prognosis. Heterogeneity analysis using the Q test, and *P* < 0.1 was considered statistically significant. The heterogeneity was evaluated according to I^2^. When I^2^ was 0–50%, it showed no or moderate heterogeneity, and when I^2^ > 50%, it showed significant heterogeneity. According to the I^2^ and *P* values, different effect models were used. When I^2^ > 50%, or *P* < 0.1, a random effects model was used. Otherwise we used a fixed effect model when the heterogeneity was low or there was no heterogeneity. Begg’s test and Egger’s test were used to determine if there was a potential publication bias in the selected studies. Sensitivity analysis was used to assesse the stability of results by excluding one study at a time. All statistical analysis and data generation were done using STATA software (StataMP 14, USA).

## Results

### Description of selected studies

Figure 1 shows our literature search and screening strategy. After removing 613 duplicate studies, a total of 4739 studies were further explored for the title and abstract. A total of 4657 studies were excluded due to non-conformity or irrelevant topics. 82 studies conducted further full-text evaluations, 35 of which were excluded due to lack of HR information on HR and 95% Cl, 16 studies were excluded because of detected IDO levels in the serum. Therefore, the final 31 studies included a total of 3939 patients for meta-analysis to analyze the association between IDO expression and prognosis in solid tumor patients [14–44].

The 31 studies included in this meta-analysis were derived from 10 countries, 6 studies originating from Europe (respectively from Belgium, Netherlands, Poland, Croatia and Germany), 18 from Asia (10 from China; and 8 from Japan), 2 from Africa (Tunisia), 3 from USA, 2 from Australia. All of these studies were published between 2006 and 2019. As for the cancer types, among the studies, esophageal cancer was the most common type of cancer (*n* = 4), followed by endometrial cancer, colorectal cancer, melanoma, and vulvar squamous cell carcinoma (*n* = 2). Other tumor types were involved in one study each. Since PFS, DFS and RFS are similar outcome endpoints, we used TTP to represent them in this meta-analysis. In these studies, 3 studies used polymerase chain reaction (qRT-PCR) to detect IDO expression in tumor tissues, while the other 28 studies used immunohistochemistry (IHC) staining to detect IDO expression. 28 datasets had information on OS, and 14 had information on TTP (PFS /DFS). According to NOS tool, we systematically evaluated the quality of the included studies, and all of these studies had high quality and the NOS scores were between 6 and 9 points. (Table 1).

**Table 1:** Characteristics of the patients included in the meta-analysis

Study	Year	Country	Cancer type	Case (n)	Age (Median/Mean, years)	Tumor stage (I/II/III/IV)	Follow-up (Median/Mean, months)	Study type	Method	Cut off value	Endpoints	NOS
Gerald. et al	2006	Austria	Colorectal cancer	143	NA	29/24/78/12	51.8^a^	Retrospective	IHC	High expression: score (5–12) Low expression: score (0–4)	OS	8
K. et al	2006	Japan	Endometrial cancer	80	57.2^a^	54/10/10/6	71.6^b^	Retrospective	IHC	High expression: score (4–6) Low expression: score (0–3)	OS, PFS	8
Rainer. et al	2007	Japan	Renal cell carcinoma	55	NA	22/33	NA	Retrospective	qPCR	High expression: Above the 80th percentile	OS	6
Ke. et al	2008	China	Hepatocellular carcinoma	138	NA	NA	NA	Retrospective	IHC	High expression: score (5–9) Low expression: score (0–4)	OS	8
Kazuhiko. et al	2008	Japan	Endometrial Cancer	65	57.7^a^	44/6/9/6	72^b^	Retrospective	IHC	High expression: score (4–5) Low expression: score (0–3)	OS, PFS	8
Hiroshi. et al	2009	Japan	Osteosarcoma	47	15^b^	0/47/0/0	67.4^b^	Retrospective	IHC	High expression: score (4) Low expression: score (0–3)	OS	7
Tomoko. et al	2010	Japan	Cervical cancer	112	NA	67/45/0/0	NA	Retrospective	IHC	High expression: > 50% of tumor cells were stained	OS, PFS	7
Jacek. et al	2011	Poland	Vulvar squamous cell carcinoma	76	69.5^b^	NA	51.23^b^	Retrospective	IHC	> 50% of tumor cells were stained with clusters of higher intensity of expression	OS	8
Reinhart. et al	2011	Belgium	Melanoma	116	52^b^	NA	71^b^	Prospective	IHC	Almost none/weak versus strong IDO expression	OS, PFS	9
Renske. et al	2012	Netherland	Endometrial carcinoma	355	64^b^	196/58/77/44	63.6^b^	Prospective	IHC	High expression: score (4–6) Low expression: score (0–3)	DFS	8
Jin. et al	2013	China	Laryngeal squamous cell carcinoma	187	52.4^b^	20/58/88/21	48.56^a^	Retrospective	IHC	High expression: score (3–4) Low expression: score (0–2)	OS, DFS	9
Yunlong. et al	2015	China	Esophageal squamous cell cancer	196	54^b^	113(I–II)/83(III–IV)	NA	Prospective	IHC	High expression: score (5–12) Low expression: score (0–4)	OS	8
Maciej. et al	2015	Poland	Melanoma	48	56.9^b^	NA	30.3^b^	Retrospective	IHC	High expression: score > 47.39 Low expression: score ≤ 47.39	OS	6
Ahlem. et al	2016	Tunisia	Nasopharyngeal carcinoma	71	NA	10(I–II)/53(III–IV)	30^b^	Prospective	IHC	High expression: score (4–5) Low expression: score (0–3)	OS, PFS	7
Hao. et al	2016	China	Gastric adenocarcinoma	357	60.3^a^	80/79/198/0	41^b^	Retrospective	IHC	With the X-tile software, the cut-off point was 282, 51% patients were separated into the IDO high expression subgroup	OS	7
Tao. et al	2017	China	Pancreatic cancer	80	NA	10(I–II)/53(III–IV)	40^b^	Prospective	IHC	High expression: score (> 4) Low expression: score (≤4)	OS	8
Tvrtko. et al	2017	Croatia	Bladder carcinomas	74	65.3^a^	NA	NA	Prospective	qPCR	IDO-positive group, in which expression of IDO gene was detected, regardless of the level of expression.	OS	7
Daniel. et al	2017	USA	Breast cancer	362	NA	278(I–II)/63(III–IV)	NA	Retrospective	IHC	Median cut-point was used to stratify IDO1 scores in low and high statuses.	OS	8
Lijie. et al	2017	USA	Glioblastoma	148	NA	NA	NA	Prospective	qPCR	IDO1 mRNA levels were stratified into IDO1- low and -high expressing groups based on the determined cutoff values.	OS	8
Wenjuan. et al	2018	China	Colorectal cancer	95	NA	NA	NA	Retrospective	IHC	High expression: score (2–3) Low expression: score (0–1)	OS	7
Yufeng. et al	2018	Taiwan (China)	Thymic carcinoma	69	54^a^	1/3/45/20	46^b^	Retrospective	IHC	High expression: score (2–3) Low expression: score (0–1)	OS, PFS	8
Hiroto. et al	2018	Japan	Esophageal cancer	182	66.5^a^	69/63/41/9	NA	Retrospective	IHC	High expression: score (2–3) Low expression: score (0–1)	RFS	7
Yuki. et al	2018	Japan	Esophageal Cancer	305	66^a^	123/80/102/0	44.4^b^	Prospective	IHC	(0; no expression, 1; weak expression, 2; moderate expression or 3; strong expression)	OS	9
Masaaki. et al	2018	Japan	Gastric Cancer	60	67.8^a^	0/0/60/0	41^a^	Retrospective	IHC	A total score of greater than 4+ was defined as IDO positive expression	OS, DFS	8
Tamkin. et al	2019	Australia	Malignant pleural mesothelioma	67	65^b^	NA	NA	Retrospective	IHC	Negative Positive (> 0%)	OS	7
Wenjuan. et al	2019	China	Adenosquamous Lung Carcinoma	183	58^b^	52/41/71/19	NA	Retrospective	IHC	High- and low-expression based on the determined cutoff values.	OS	8
Devarati. et al	2019	USA	Anal cancer	63	61^b^	7/24/9/21 (2 unknown)	35^b^	Retrospective	IHC	Positive (> 50% IDO1 expression)	OS	8
Julia. et al	2019	Germany	Rectal cancer	91	64^b^	NA	NA	Retrospective	IHC	High expression: score (3–6) Low expression: score (0–2)	OS, DFS	8
Nadia. et al	2019	Tunisia	Vulvar squamous cell carcinoma	61	65.61^a^	29/4/26/2	NA	Retrospective	IHC	High expression: score (3) Low expression: score (0–2)	OS, DFS	7
Sha. et al	2019	China	Esophageal squamous cell carcinoma	158	56^b^	0/34/124/0	40.2^b^	Retrospective	IHC	Positive (> 50% IDO1 expression)	RFS	8
Yuhshyan. et al	2019	Taiwan (China)	Bladder cancer	108	68^a^	45/43/19/1	45^b^	Retrospective	IHC	Strongly Positive (> 25% IDO1 expression)	OS, PFS	8

Abbreviations: *IHC* Immunohistochemistry, *qPCR* Quantitative Real Time Polymerase Chain Reaction, *NOS* Newcastle-Ottawa Scale, *OS* overall survival, *DFS* disease free survival, *PFS* progression free survival. ^a^ Mean, ^b^ Median. NA: Not Available

### Impact of IDO expression on cancer prognosis

**Fig. 2:**
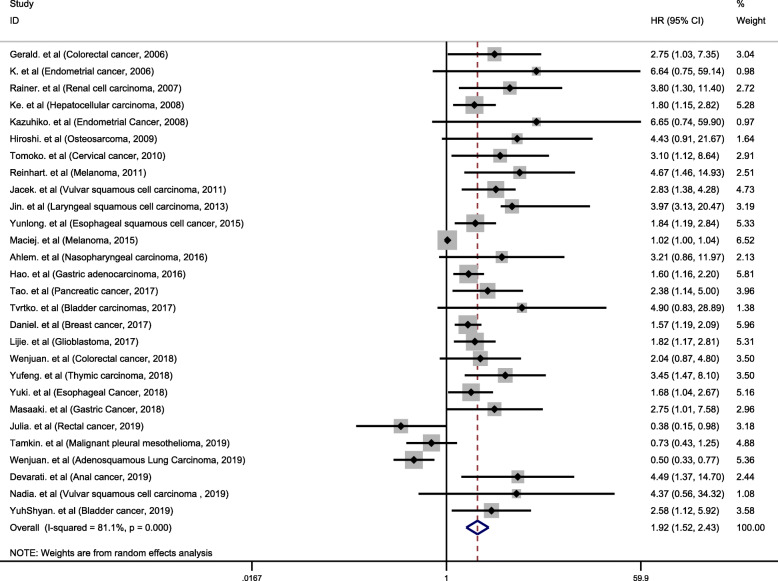
Meta-analysis of impact of IDO expression on prognosis of patients with solid tumors. Forest plot of HRs for correlation between IDO expression and OS in solid tumor patients. Results are presented as individual and metaHR, and 95% CI. The random-effects model was used. The square size of individual studies represented the weight of the study. Vertical lines represent 95% CI of the pooled estimate. The diamond represents the overall summary estimate, with the 95% CI given by its width

**Fig. 3:**
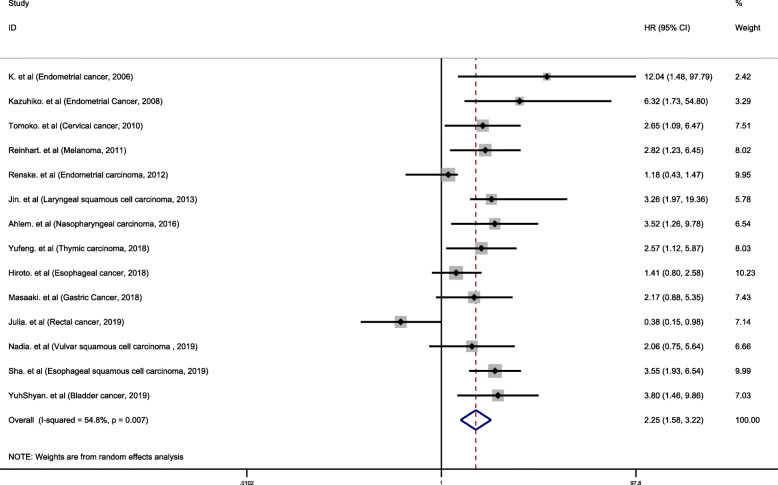
Forest plot of HRs for correlation between IDO expression and TTP in solid tumor patients. Results are presented as individual and metaHR, and 95% CI. The random-effects model was used. The square size of individual studies represents the weight of the study. Vertical lines represent 95% CI of the pooled estimate. The diamond represents the overall summary estimate, with the 95% CI given by its width

In the included studies, a total of 28 studies analyzed the association between IDO expression and OS. Of these 28 studies, 3 studies with HR < 1 [38, 39, 41], and 18 studies with HR > 2 [14–16, 18–22, 24, 27, 29, 30, 33, 34, 37, 42–44]. We performed a meta-analysis of 28 studies. Since I^2^ values was 81.1%, the random effects model was used to calculate the pooled HR and 95% CI. The combined analysis of 28 datasets indicated that compared with IDO negative/low expression, IDO positivity/high expression was highly correlated with poor prognosis in cancer patients (pooled HR 1.92, 95% CI 1.52–2.43, *P* < 0.001) (Fig. 2). A total of 14 studies were used to assess the association between IDO expression and TTP. We calculated the pooled HR using a random effects model, because the heterogeneity test indicated an I^2^ value of 54.8% and a *P* value of 0.007. The results indicated that high expression of IDO was highly correlated with poor prognosis of TTP (pooled HR = 2.25, 95% CI 1.58–3.22, *P* < 0.001) (Fig. 3).

### Subgroup analysis

Since the results from the meta-analysis indicated significant heterogeneity, we performed heterogeneity analysis in order to identify potential factors that may cause heterogeneity. We classified the included studies and performed heterogeneity analysis based on study location, detection method, sample size, study type, cancer type, age, follow-up periods and study quality. Subgroup analysis showed that the high expression of IDO was highly correlated with poor OS and TTP, but the heterogeneity was not significantly reduced according to different study locations, detection method, sample size grouping, average age and study quality. However, in a prospective study group, we found that high expression of IDO was highly correlated with poor OS prognosis (HR1.98, 95% CI 1.57–2.49, *P* < 0.001) and there was no heterogeneity (I^2^ = 0%, *P* = 0.6) (Table 2). Subgroup analysis showed that there was no heterogeneity among bladder cancer, colorectal cancer, endometrial cancer and esophageal cancer studies. Heterogeneity was also significantly reduced among studies of the same type of tumor, such as digestive system tumors and reproductive system tumors (Table 2). In addition, there was no significant heterogeneity (HR 3.41, 95% CI 2.41–4.83, *P* < 0.001. I^2^ = 0%, *P* = 0.97) between studies with an average follow-up period of more than 45 months (Table 2).

**Table 2:** Hazard ratio for the association between IDO overexpression and solid tumors prognosis

Stratified analysis	Effect size	NO. of study	Cases	HR		Heterogeneity	
				Pooled HR (95% CI)	*P* value	*I*^2^ (%)	*p* value
**All studies**							
OS	OS	28	3457	1.92 (1.52–2.43)	< 0.001	81.1	< 0.001
TTP	TTP	14	1815	2.25 (1.58–3.22)	< 0.001	54.8	0.007
**Study location**							
Asia	OS	16	2137	2.12 (1.54–2.92)	< 0.001	68.5	< 0.001
	TTP	9	1121	2.48 (1.74–3.55)	< 0.001	11.4	0.342
Other countries	OS	12	1320	1.66 (1.17–2.37)	0.005	82.2	< 0.001
	TTP	5	694	1.99 (1.32–2.98)	0.001	14.3	0.323
**Detection method**							
IHC	OS	25	3180	1.86 (1.46–2.38)	< 0.001	81.3	< 0.001
	TTP	14	1815	2.25 (1.58–3.22)	< 0.001	54.8	0.007
qPCR	OS	3	277	2.11 (1.42–3.13)	< 0.001	17.7	0.297
**Sample size**							
< 70	OS	9	535	2.25 (1.31–3.88)	0.003	75.5	< 0.001
	TTP	4	255	2.49 (1.51–4.10)	< 0.001	0.0	0.72
70–120	OS	10	903	2.37 (1.42–3.95)	0.001	55.9	0.02
	TTP	6	578	2.43 (1.09–5.44)	0.03	72.8	0.003
> 140	OS	9	2019	1.60 (1.18–2.18)	0.003	75.8	< 0.001
	TTP	4	882	1.98 (1.12–3.51)	0.019	63.2	0.043
**Study type**							
Retrospective	OS	21	2807	1.82 (1.39–2.40)	< 0.001	81.5	< 0.001
	TTP	11	1273	2.32 (1.50–3.60)	< 0.001	57.9	0.008
Prospective	OS	7	650	1.98 (1.57–2.49)	< 0.001	0	0.6
	TTP	3	542	2.09 (1.03–4.23)	0.04	56.2	0.102
**Cancer type**							
Digestive system tumor	OS	10	1528	1.79 (1.38–2.31)	< 0.001	40.8	0.085
Reproductive system tumor	OS	6	756	2.39 (1.53–3.72)	< 0.001	34.9	0.175
Bladder cancer	OS	2	182	2.90 (1.32–6.15)	0.006	0.0	0.521
Colorectal cancer	OS	2	238	2.32 (1.22–4.42)	0.01	0.0	0.655
Endometrial cancer	OS	2	145	6.64 (1.41–31.27)	0.017	0.0	0.99
Esophageal cancer	OS	2	501	1.76 (1.28–2.43)	0.001	0.0	0.79
Esophageal cancer	TTP	2	340	2.23 (0.91–5.49)	0.081	77.9	0.033
Gastric Cancer	OS	2	417	1.68 (1.22–2.32)	0.001	1.5	0.314
Melanoma	OS	2	164	1.95 (0.45–8.49)	0.376	84.8	0.01
Vulvar squamous cell carcinoma	OS	2	137	2.92 (1.69–5.04)	< 0.001	0.0	0.69
**Age (Mean/Median)**							
< 60 years	OS	9	991	2.02 (1.22–3.36)	0.007	83.6	< 0.001
> 60 years	OS	10	1262	1.76 (1.16–2.67)	0.008	68.8	0.001
**Follow-up (Median/Mean)**							
≤ 45 months	OS	8	1092	1.90 (1.29–2.78)	0.001	79.4	< 0.001
> 45 months	OS	8	783	3.41 (2.41–4.83)	< 0.001	0.0	0.97
**Study quality**							
NOS score > 7	OS	18	2825	2.00 (1.48–2.69)	< 0.001	72.6	< 0.001
NOS score ≤ 7	OS	10	632	1.75 (1.20–1.57)	< 0.001	72.4	< 0.001

Abbreviations: *HR* hazard ratio, *CI* confidence interval, *OS* overall survival, *TTP* time to tumor progression, *IHC* Immunohistochemistry, *qPCR* Quantitative Real Time Polymerase Chain Reaction

### Publication bias and sensitivity analysis

**Fig. 4:**
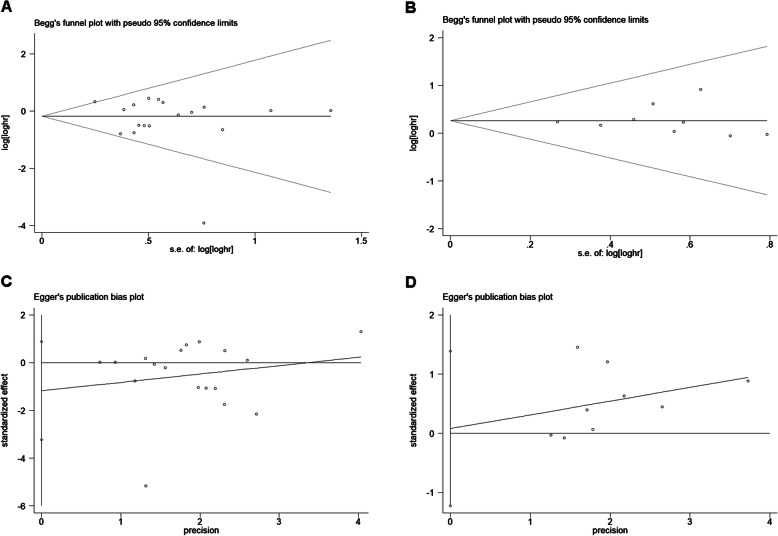
Begg’s funnel plots and Egger’s publication bias plots for studies involved in the meta-analysis. Begg’s funnel plots for the studies included in meta-analysis regarding. OS (**a**) and TTP (**b**). Each hazard ratio (HR) was plotted on an HR scale against its standard error (SE). The horizontal lines indicate the pooled estimate of the overall HR, with the sloping lines reflecting the expected 95% confidence interval for a given SE. Egger’s publication bias plots for the studies included in meta-analysis regarding OS (**c**) and TTP (**d**). The 95% confidence intervals of the regression line’s y intercept include zero, *P* values were 0.59 and 0.89, respectively, indicating that there was no evidence of publication bias

**Fig. 5:**
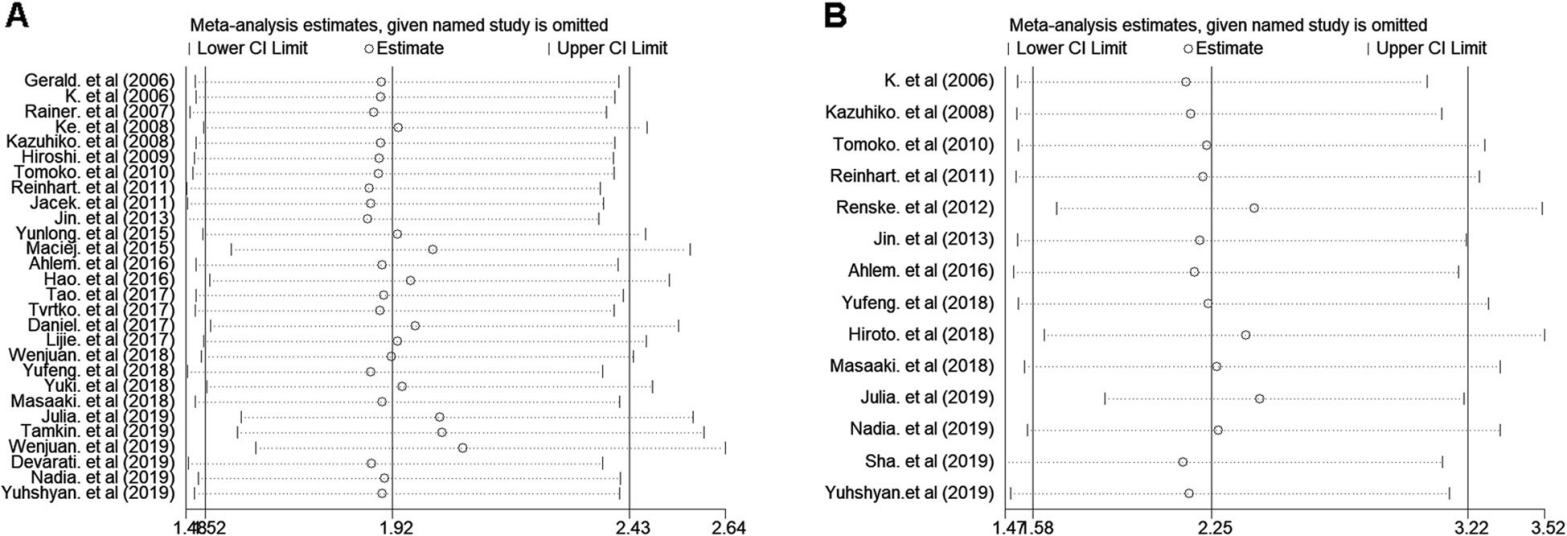
Sensitivity analysis of the meta-analysis. **a** Overall survival. **b** Time to tumor progression. The vertical axis at 1.98 and 2.25 indicates the overall HR, and the vertical lines on either side of 1.98 and 2.25 indicate the 95% CI. Every hollow round indicates the pooled HR when the left study was omitted in a meta-analysis with a random model. The two ends of every broken line represent the respective 95% CI

Evaluation of publication bias between studies was done using Begg’s funnel plot and Egger’s test. The shape of the OS and TTP funnel plots were not significantly asymmetrical, and the Egger’s test indicated OS (*P* = 0.47) and TTP (*P* = 0.89). These results suggested that there was no significant publication bias in the meta-analysis of IDO expression in relation to OS and TTP prognosis (Fig. 4). Sensitivity analysis refers to the removal of a study each time to analyze the impact of individual studies on the stability of meta-analysis results. Sensitivity analysis showed that no single study had a significant impact on the conclusions of this meta-analysis (Fig. 5).

## Discussions

In this study, we systematically assessed IDO expression level and prognostic indicators of 3939 solid tumor patients from 31 different studies. Our results showed that high expression of IDO predicted poor OS and TTP in cancer patients. However, the results from this meta-analysis indicated that there was significant heterogeneity among these studies. The Begg’s funnel plot and Egger’s test showed that there was no significant publication bias in this meta-analysis, and the sensitivity analysis showed that no single study can influence the conclusion of this meta-analysis.

High expression of IDO was highly correlated with poor prognosis of OS and TTP. However, the heterogeneity was also obvious. It was not difficult to understand that there will be heterogeneity in our study. In 31 studies, a total of 10 tumor types were included, and the role of IDO in different tumors may be inconsistent. For example, three studies have concluded to the contrary. In addition, the study type, IDO test method, number of patients included, follow-up period, and study quality were different in each study, all these factors can lead to heterogeneity. To this end, we performed a subgroup analysis to explore the source of heterogeneity. Subgroup analysis showed that the study location, sample size, and age were not sources of heterogeneity. For OS, no heterogeneity in prospective studies and follow-up period over 45 months studies. These results indicate that the type of study and follow-up period were the reasons for the heterogeneity in this meta-analysis. In addition, in the same type of tumor research (such as digestive system tumors and reproductive system tumors), there was no obvious heterogeneity. Subgroup analysis also showed no heterogeneity in bladder cancer, colorectal cancer, endometrial cancer and esophageal cancer, gastric cancer and vulvar squamous cell carcinoma studies. The difference in study quality may also be the cause of heterogeneity. To this end, we used the NOS score to evaluate the quality of each study and performed a subgroup analysis based on the NOS score. We found that the high-scoring study group did not significantly reduce heterogeneity. Therefore, in this meta-analysis, the quality of study is not the main reason for heterogeneity.

Our study further enhanced the view that high expression of IDO has a poor prognosis for cancer patients by performing meta-analysis on a large number of research data. In addition, this meta-analysis also gives hints on several other aspects. First, the high expression of IDO may be a universal prognostic biomarker for solid tumors. We analyzed 10 different types of solid tumors, including colorectal cancer, endometrial cancer, renal cell carcinoma, hepatocellular carcinoma, etc. Secondly, we verified that both Asian patients and other country patients harboring high expression of IDO were highly correlated with poor prognosis in patients with solid tumors, which did not vary because of ethnic differences. Moreover, our results suggested that the IDO expression can be used as a more widely prognostic biomarker. Finally, this study suggested that IDO had the potential to develop into a prognostic biomarker and a therapeutic target for solid tumors.

It should be noted that, there were limitations in this meta-analysis. First, the definitions of IDO positive and high expression were not completely consistent between studies, which may cause heterogeneity between studies. Secondly, due to limitations from the other included studies and large number of tumor types, we were unable to perform a subgroup analysis for each type of tumor. Thirdly, we extracted the HRs data directly from the original literature, and these data were reliable than calculated HRs indirectly deducted from the literature. However, some studies did not provide complete data and were excluded from statistics, hence some missing information might have reduced the power of IDO as a prognostic biomarker in solid tumor patients.

## Conclusions

In summary, this meta-analysis clearly demonstrated that the high expression of IDO in tumor tissues was closely related to poor survival of tumor patients. Our study suggested that IDO may be used as a potential tumor prognostic biomarker and tumor treatment target.

## Data Availability

All data generated or analyzed during this study are included in this published article. The datasets used and/or analysed during the current study available from the corresponding author on reasonable request.

## Abbreviations

IDO: Indoleamine 2,3-dioxygenase
OS: Overall survival
TTP: Time to progression
HR: Hazard ratio
CI: Confidence interval
Tregs: Regulatory T-cells
1-MT: 1-methyltryptophan
DSS: Disease-specific survival
RFS: Relapse-free survival
DFS: Disease-free survival
TTR: Time to recurrence
NOS: Newcastle-Ottawa Scale
